# Galacto-Oligosaccharides Preserve the Colonic Mucus Barrier Against Dietary Advanced Glycation End Products Through Modulation of Mucin Glycosylation and ERK Signaling

**DOI:** 10.3390/foods15213806

**Published:** 2026-10-23

**Authors:** Chenxi Nie, Xiaojin Yuan, Huicui Liu, Qingyu Ma, Baoming Tian, Jing Li, Min Zhang, Zhifei Chen, Juxiu Li

**Affiliations:** 1 College of Food Science and Engineering, Bohai University, National & Local Joint Engineering Research Center of Storage, Processing and Safety Control Technology for Fresh Agricultural and Aquatic Products, China Light Industry Key Laboratory of Marine Fish Processing, Jinzhou 121013, China; 2 College of Food Science and Engineering, Northwest A&F University, 22 Xinong Road, Yangling 712100, China; 3 Hainan Institute of Northwest A&F University, Sanya 572024, China

**Keywords:** advanced glycation end products (AGEs), galacto-oligosaccharides (GOS), mucus barrier, goblet cells, glycosyltransferases

## Abstract

Advanced glycation end products (AGEs) are prevalent in thermally processed foods. Long-term intake of AGEs has been linked to intestinal barrier damage, contributing to the development of chronic diseases. Galacto-oligosaccharides (GOS) restored mucus barrier damage induced by dietary AGEs, but the underlying mechanisms remained unclear. This study focused on investigating the effects of GOS on mucin synthesis-related glycosyltransferases, as well as differentiation and apoptosis of goblet cells (GCs) in dietary AGEs-fed C57BL/6 mice. Fecal microbiota transplantation (FMT) was used to probe a possible contribution of the intestinal microbiota. The findings demonstrated that GOS upregulated the mRNA expression of glycosyltransferases (St3gal1 and St3gal3) while inhibiting p-ERK signaling. In addition, no marked changes in mRNA expression of the Wnt, BMP, Notch, and CHOP pathways (which are involved in GC proliferation, differentiation, and apoptosis) were observed following GOS intervention. FMT data suggested the hypothesis that the gut microbiota may participate in mediating changes in St3gal1 and p-ERK/ERK in response to GOS. These findings provided valuable insights into potential interventions for intestinal mucus barrier damage induced by AGEs.

## 1. Introduction

The increasing consumption of thermally processed foods has raised concerns regarding the health effects of dietary advanced glycation end products (AGEs), a heterogeneous group of compounds generated primarily through nonenzymatic reactions between reducing sugars and free amino groups during food processing [1,2]. Accumulating evidence has linked chronic exposure to dietary AGEs with metabolic and microvascular disorders, including chronic kidney disease [1]. Importantly, the gastrointestinal tract is among the first biological systems to encounter dietary AGEs, suggesting that intestinal tissues may represent an early target of AGEs-associated toxicity. 

A substantial proportion of ingested dietary AGEs resists gastrointestinal digestion and reaches the colon [3], where they may directly interact with the intestinal mucosa and resident microbiota [1,4]. The colonic barrier is a multilayered defense system consisting of the mucus layer (covering the colonic intestinal epithelium), the epithelial glycocalyx (on the microvilli of absorptive intestinal epithelial cells), and intercellular junctions, including tight and adherens junctions [5]. Previous studies of AGEs-induced intestinal barrier dysfunction have predominantly focused on epithelial junctional proteins, such as zonula occludens-1 (ZO-1), occludin, and claudins, as well as systemic markers of barrier disruption, including circulating lipopolysaccharide (LPS) [1,6]. However, the mucus layer constitutes the first physical interface between luminal contents and the intestinal epithelium and therefore represents a critical [5], yet comparatively underexplored, component of the intestinal barrier.

The colonic mucus layer is primarily composed of highly glycosylated mucins secreted by goblet cells (GCs), with mucin O-glycosylation being essential for the structural organization, hydration, and protective properties of the mucus network [7,8,9]. Our previous intervention study showed that chronic consumption of an AGEs-enriched diet impaired the colonic mucus barrier, as evidenced by reduced goblet cell abundance and mucin production, and that these alterations occurred before detectable disruption of epithelial junctional integrity [4]. Notably, dietary supplementation with galacto-oligosaccharides (GOS), a well-characterized prebiotic, substantially alleviated AGEs-induced mucus barrier damage [4]. However, the mechanism by which GOS supplementation ameliorates the colonic mucus barrier under chronic dietary AGE exposure remains unclear.

Maintenance of the mucus barrier depends on both goblet cell homeostasis and the biosynthesis and post-translational modification of mucins [10,11,12]. Goblet cell abundance is controlled by a coordinated balance among intestinal stem cell proliferation, lineage differentiation, and programmed cell death [13,14]. The Wnt and BMP pathways regulate intestinal epithelial proliferation, whereas Notch signaling is a major determinant of secretory lineage differentiation, including goblet cell development [15,16]. In addition, apoptosis and cellular stress pathways, including C/EBP homologous protein (CHOP)-associated signaling and mitogen-activated protein kinase (MAPK) pathways, may influence goblet cell survival [15,17]. Therefore, alterations in goblet cell number could theoretically contribute to AGEs-induced mucus barrier dysfunction and its amelioration by GOS.

In parallel, mucus barrier function is determined not only by the quantity of mucin produced but also by its glycan composition and structural organization [9]. Mucin biosynthesis requires the coordinated action of multiple glycosyltransferases, which regulate glycan chain initiation, extension, branching, and termination (including fucosyltransferases and sialyltransferases) [18]. These glycan modifications influence important physicochemical properties of mucus, including viscosity, hydration, stability, and interactions with intestinal microorganisms [9]. However, whether dietary AGEs alter the expression of mucin glycosylation-related enzymes and whether GOS restores these alterations remain largely unknown.

The molecular mechanisms through which dietary AGEs disrupt the mucus barrier may additionally involve AGEs-associated cellular signaling. The receptor for advanced glycation end products (RAGE), a major cellular receptor for AGEs, is widely expressed in the gastrointestinal tract and has been implicated in intestinal barrier dysfunction [19]. AGE-RAGE signaling can activate downstream intracellular pathways, including MAPK signaling [20], which may influence epithelial homeostasis and mucosal responses. Increasing evidence further suggests that the gut microbiota is an important determinant of host responses to dietary AGEs [21]. Dietary AGEs can modify the intestinal microbial ecosystem, whereas microbiota-derived metabolites and microbial-mucosal interactions can in turn regulate epithelial integrity and mucus homeostasis [1,4,21,22]. Importantly, our previous fecal microbiota transplantation (FMT) study demonstrated that transplantation of microbiota from GOS-treated mice partially reproduced the protective effects of GOS on the colonic mucus barrier in AGEs-fed mice [4]. These findings provide preliminary evidence that the gut microbiota may act as an intermediary through which GOS regulates the host mucosal response to dietary AGEs. Nevertheless, the specific molecular pathways linking GOS-modulated microbiota to mucin barrier homeostasis remain unclear.

Based on these observations, we hypothesized that GOS protects against dietary AGEs-induced colonic mucus barrier dysfunction by modulating goblet cell homeostasis and/or mucin glycosylation, potentially through a gut microbiota-dependent signaling mechanism. To test this hypothesis, we first examined the effects of GOS on mucin production and the expression of mucin glycosylation-related enzymes in the colonic mucosa of AGEs-fed mice. We then evaluated key signaling pathways involved in goblet cell proliferation, differentiation, and apoptosis, including Wnt, BMP, and Notch, as well as CHOP and MAPK signaling. Finally, FMT was used to explore whether GOS-associated effects on mucin glycosylation and ERK signaling might be transmissible via fecal materials. This study aims to investigate the underlying mechanisms of the protective effects of GOS against diet AGEs-induced dysfunction of the colonic mucosal barrier, which may provide a foundation for new strategies to prevent AGEs-induced colonic barrier damage.

## 2. Materials and Methods

### 2.1. Chemicals and Reagents

GOS (purity > 90%) was provided by Baolingbao Biology (Shandong, China). The degree of polymerization (DP) of its oligosaccharide components ranged from DP2 to DP8. On a dry weight basis, the residual components comprised <10% anhydrous lactose and <3% anhydrous glucose. FastKing RT Kit was purchased from Tiangen Biotech (Beijing, China). AG RNAex Pro reagent and SYBR Green Premix Pro Taq HS qPCR kit were purchased from Accurate Biology (Changsha, China). The whole Protein Extraction Kit was purchased from Beijing Solarbio Science and Technology Co., Ltd. (Beijing, China). The anti-Bax, anti-GRP78, anti-Bcl-2, anti-JNK, anti-p-JNK, anti-p38, anti-p-p38, anti-ERK1/2, anti-p-ERK1/2, and anti-MyD88 were purchased from Wanlei Biologicals (Shenyang, China). ECL Chemiluminescent Substrate Reagent Kit was purchased from ZHHC Biotechnology Co. (Xi’an, China). The anti-GAPDH antibody was purchased from Huabio (Hangzhou, China). We purchased the secondary antibody (DyLight 594-conjugated goat anti-rabbit IgG) and DAPI from Abbkine Scientific Co., Ltd. (Wuhan, China).

### 2.2. Animals and Experimental Design I, Donor Mice

A total of 24 male C57BL/6 mice at five weeks of age were obtained from Xi’an Ensiweier (Xi’an, China) and were kept in controlled environmental conditions (light/dark cycle of 12/12 h, temperature of 22 ± 2 °C, humidity of 55–65%). The present study reused the identical diet and the same C57BL/6 mice from our previous study [4]. Briefly, after 7 days of adaptation, C57BL/6 mice were randomly divided into 3 groups (n = 8): the normal control fed with AIN93G diet (Con), the AGEs group fed with AIN93G heated at 170 °C for 45 min (AGE), and the AGE + GOS (500 mg/kg·BW) group (GA). The dosage of 500 mg/kg·BW refers to the mass of GOS solids, not the total mass of the commercial powder. GOS was dissolved in ultrapure water for gavage administration; mice in the Con group received an equal volume of ultrapure water via daily gavage. GOS doses were selected based on reported human dietary oligosaccharide intake. Assuming that GOS accounts for approximately 25% of total dietary oligosaccharide intake, total oligosaccharide intakes of 2 and 12 g/day correspond to estimated GOS intakes of 0.5 and 3.0 g/day, respectively [23,24]. For a 70 kg adult, these amounts are equivalent to 7.14 and 42.86 mg/kg·BW per day, respectively. Using conventional body surface area normalization (Km = 37 for humans and Km = 3 for mice), the corresponding mouse-equivalent doses were estimated to be approximately 88 mg/kg·BW and 529 mg/kg·BW per day, respectively. Therefore, a GOS dose of 500 mg/kg·BW was selected based on the upper estimated mouse-equivalent dose. The animal protocol was approved by the Animal Ethics Committee of Northwest A&F University (SP2022048).

### 2.3. Animals and Experimental Design II, Recipient Mice

Five-week-old male C57BL/6 mice were acclimatized for one week prior to treatment. Subsequently, the mice were treated with an antibiotic cocktail (ABX: ampicillin 1 g L^−1^, metronidazole 1 g L^−1^, vancomycin 0.5 g L^−1^, and neomycin 0.5 g L^−1^) via drinking water for two weeks. Afterward, mice were randomly divided into three groups (designated FC, FA, and FG groups, n = 10 per group) and fed an AGEs diet. Fecal microbiota transplantation was performed according to a previously described method with slight modifications [25]. Briefly, the fresh stool samples (200 mg) from donor mice of each group were suspended in sterile saline (2 mL). The mixture was vigorously vortexed for 180 s and centrifuged at 800× *g* for 3 min. To avoid changes in bacterial constituents, fresh fecal transplant inoculum was prepared no more than 10 min before gavage. Fresh fecal supernatants from the Con, AGE, and GA donor groups were administered via gavage once daily at a dose of 200 μL per mouse to the FC, FA, and FG recipient groups, respectively. The donor and recipient mice were euthanized upon experiment termination (the 16th week and 8th week, respectively), and the colon was frozen and preserved for subsequent experimental analysis.

### 2.4. Quantitative Real-Time PCR (qPCR) 

Total RNA was isolated from colonic tissue using AG RNAex Pro reagent following the manufacturer’s instructions, and then reverse transcribed using the FastKing RT Kit. Quantitative real-time PCR was performed using the SYBR Green Premix Pro Taq HS qPCR kit and a real-time PCR system (QuantStudio 5, Applied Biosystems, Foster City, CA, USA). Results were quantified via the 2^-ΔΔCt^ formula approach [26]. The primer sequences used in the present study were listed in Table 1.

### 2.5. Western Blot Analysis

Total proteins were isolated from colonic tissues using the Whole Protein Extraction Kit according to the manufacturer’s instructions. Then, total colon proteins were loaded onto CFAS Any KD PAGE gels and transferred onto PVDF membranes (0.45 μm, Merck Millipore, Darmstadt, Germany). After blocking with 5% skimmed milk powder, the incubation was performed overnight with primary antibody, followed by incubation with an HRP-conjugated goat anti-rabbit IgG secondary antibody and visualization with ECL Chemiluminescent Substrate Reagent Kit. Finally, the PVDF membrane was exposed to a chemiluminescence imaging system (Sage Venture Technology Co., Ltd., Beijing, China). The protein bands were quantified using ImageJ 1.8.0 software. The primary antibodies used in this study are shown in Table 2.

### 2.6. Statistical Analysis 

All experimental data were expressed as mean ± standard error, and all statistical analyses were performed using SPSS V20.0 software (IBM, Armonk, NY, USA). Normality was examined by the Shapiro–Wilk test, and Levene’s test was adopted to assess homogeneity of variance. One-way ANOVA followed by Duncan’s test was applied for normally distributed data with equal variance, and non-parametric tests (Kruskal–Wallis) were used in case of heterogeneity of variance. Differences with *p* < 0.05 were regarded as statistically significant. All figures were plotted using GraphPad Prism 9.4.1 (San Diego, CA, USA).

## 3. Results

### 3.1. Effect of GOS on Glycosyltransferase Related to Mucin Glycan Chain Elongation in the Colon of Mice Fed with Dietary AGEs

Our previous study demonstrated that GOS supplementation alleviated the reduction in colonic mucin abundance in mice fed a dietary AGEs-enriched diet [4]. Because mucin structure and function are critically dependent on O-glycan biosynthesis, we first examined whether GOS restored AGEs-induced alterations in the enzymes responsible for mucin core structure formation. Mucin O-glycans are synthesized through a stepwise process involving multiple glycosyltransferases that regulate glycan initiation, extension, branching, and termination [18]. The elongation and branching of O-glycans generally fall into four main categories of structures (e.g., core 1–4) [27]. Core 2 structures predominate among colonic mucin O-glycans in mice [28].

**Figure 1:**
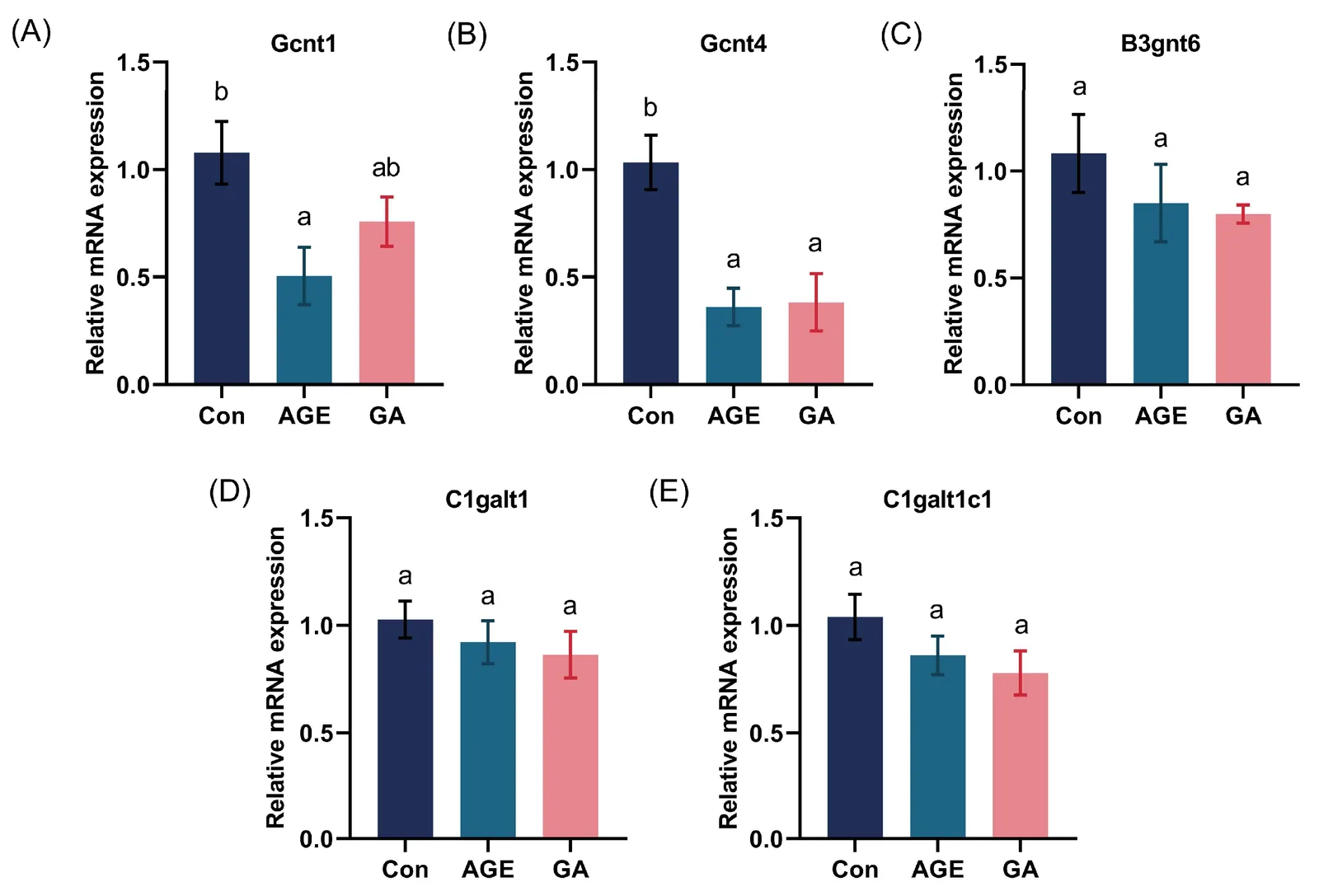
Effect of GOS on the expression of glycosyltransferases involved in glycan chain elongation in the colon of dietary AGEs-fed mice. The mRNA expression levels of Gcnt1 (**A**), Gcnt4 (**B**), B3gnt6 (**C**), C1galt (**D**), and C1galt1c1 (**E**) in the colon tissues were investigated. Different letters represent significant differences (*p* < 0.05).

As shown in Figure 1, the mRNA expression levels of Gcnt1 and Gcnt4, which are involved in the biosynthesis of core 2 O-glycans, were significantly lower in the AGE group than in the Con group (*p* < 0.05). However, GOS supplementation did not restore the expression of either Gcnt1 or Gcnt4. We further examined the mRNA expression levels of C1galt1, C1galt1c1, and B3gnt6, which encode enzymes involved in core 1 and core 3 O-glycan biosynthesis. No significant differences were detected among the Con, AGE, and GA groups (*p* > 0.05).

These findings suggested that although dietary AGE exposure altered the expression of core 2-associated glycosyltransferases, the protective effect of GOS on colonic mucin abundance was not associated with restored mRNA expression of the major mucin core structures.

### 3.2. Effect of GOS on Glycosyltransferase Related to Mucin Glycan Chain Termination in the Colon of Mice Fed with Dietary AGEs

Because GOS did not restore the mRNA expression of genes encoding enzymes involved in mucin core structure formation, we next investigated whether GOS affected the terminal modification of mucin O-glycans. Fucosyltransferases and sialyltransferases participate in the terminal modification of O-glycan chains, contributing to the formation of glycan structures that influence mucus properties and host-microbe interactions [28].

**Figure 2:**
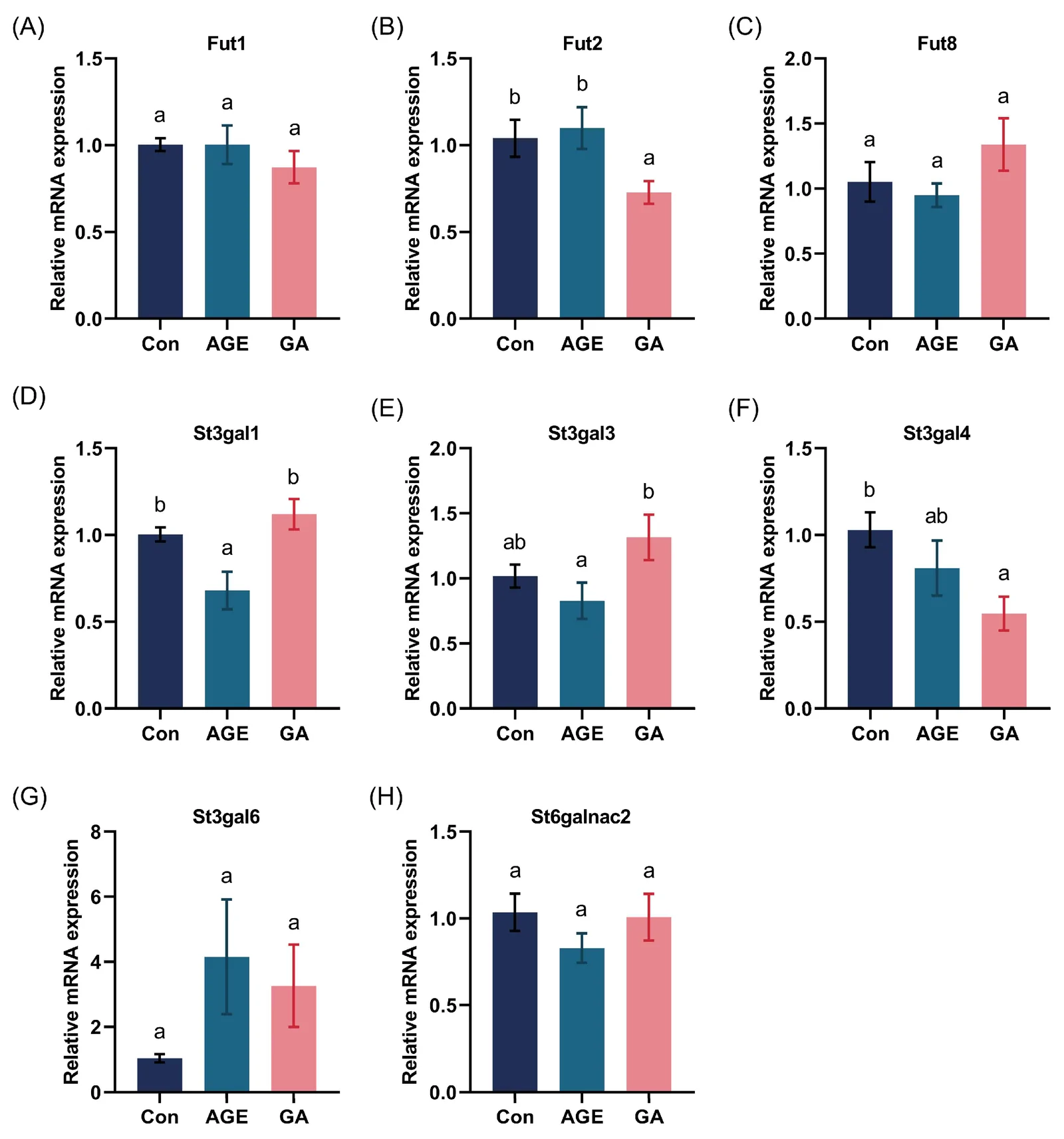
Effect of GOS on the expression of glycosyltransferases involved in mucin glycan chain termination in the colon of dietary AGEs-fed mice. The mRNA expression levels of Fut1 (**A**), Fut2 (**B**), Fut8 (**C**), St3gal1 (**D**), St3gal3 (**E**), St3gal4 (**F**), St3gal6 (**G**), and St6galnac2 (**H**) in the colon tissues were investigated. Different letters represent significant differences (*p* < 0.05).

As shown in Figure 2A–C, the expression of Fut1, Fut2, and Fut8 did not differ significantly between the Con and AGE groups. However, Fut2 expression was significantly lower in the GA group than in both the Con and AGE groups (*p* < 0.05). Among the sialyltransferases examined (Figure 2D–H), St3gal1 expression was significantly reduced in the AGE group compared with the Con group (*p* < 0.05), whereas GOS supplementation significantly increased St3gal1 expression compared with the AGE group (*p* < 0.05). In contrast, St3gal3, St3gal4, St3gal6, and St6galnac2 did not differ significantly between the Con and AGE groups. Notably, GOS supplementation significantly increased St3gal3 expression compared with the AGE group (*p* < 0.05), whereas no significant differences were detected for St3gal4, St3gal6, or St6galnac2.

Taken together, these findings suggested that the protective effect of GOS on the colonic mucus barrier was associated with selective modulation of mRNA expression related to terminal mucin glycosylation, particularly St3gal1 and St3gal3, rather than broad alterations in mRNA expression involved in overall mucin glycan biosynthesis.

### 3.3. Effects of GOS on Wnt, BMP, and Notch Signaling

Our previous study demonstrated that GOS supplementation alleviated dietary AGEs-induced reduction in colonic goblet cell abundance [4]. Goblet cell abundance is regulated by intestinal stem cell proliferation and lineage differentiation [29]; we next investigated whether GOS exerted its protective effect through the canonical Wnt, BMP, or Notch pathways.

The Wnt and BMP pathways contribute to intestinal stem cell proliferation [30], whereas Notch signaling is a key determinant of absorptive versus secretory lineage differentiation [31]. We therefore examined representative markers associated with these pathways. As shown in Supplementary Figure S1A–D and Supplementary Figure S2A–E, no significant differences were observed in the expression of Wnt-related genes (Gpr81, Wnt3, Lgr5, and Ki67) or BMP-related genes (BMP2, BMP4, SMAD1, SMAD5, and SMAD8) between the GA and AGE groups (*p* > 0.05).

**Figure 3:**
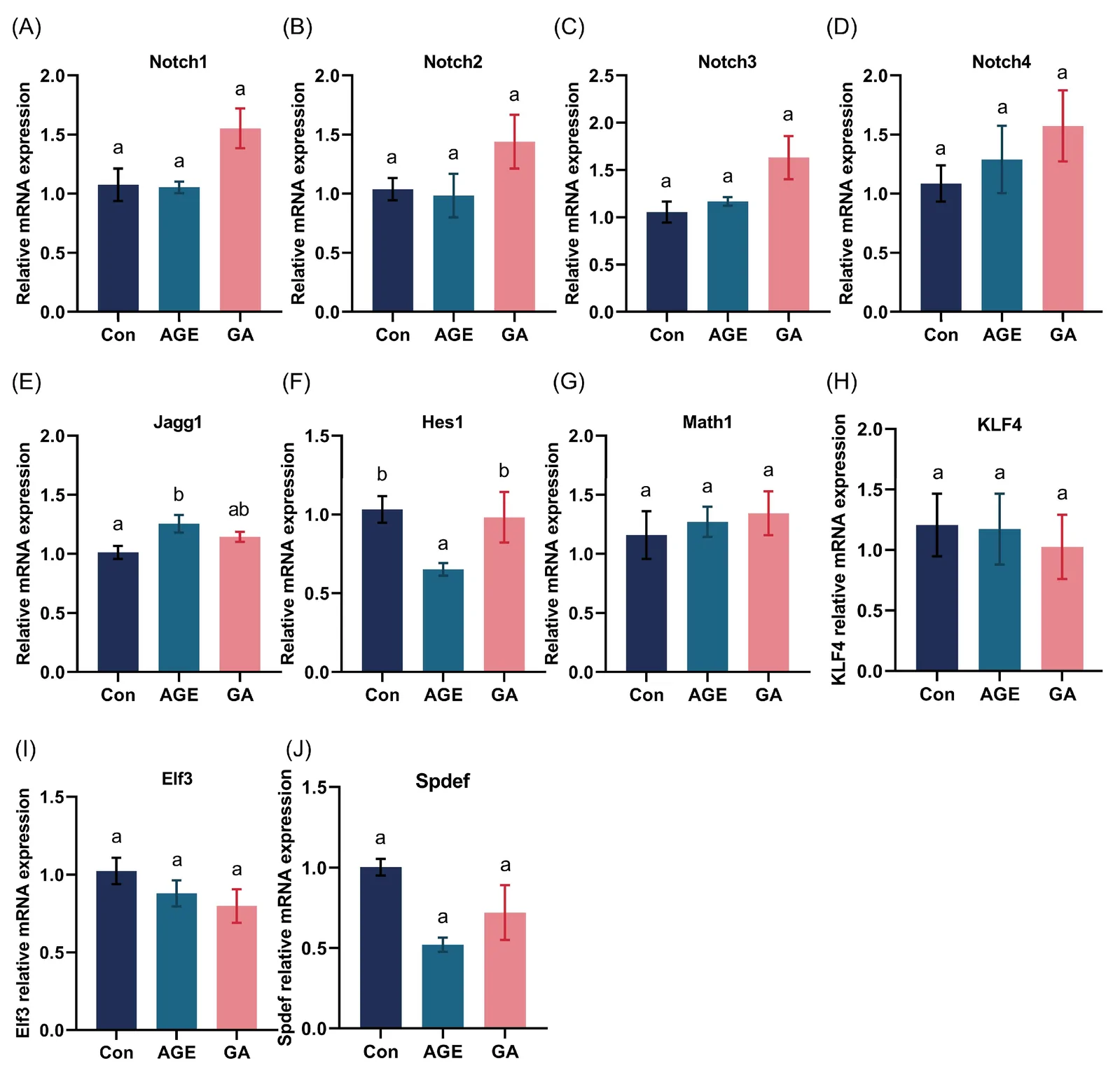
Effects of GOS on mRNA expression of genes related to goblet cell differentiation in the colon of mice fed an AGEs-diet. The mRNA expression levels of Notch1 (**A**), Notch2 (**B**), Notch3 (**C**), Notch4 (**D**), Jagg1 (**E**), Hes1 (**F**), Math1 (**G**), KLF4 (**H**), Elf3 (**I**), and Spdef (**J**) in the colon tissues were investigated. Different letters indicate significant differences (*p* < 0.05).

As shown in Figure 3A–G, no significant differences were observed in the expression of Notch-related genes (Notch1, Notch2, Notch3, Notch4 and Math1, *p* > 0.05). Consistent with these findings, GOS supplementation did not significantly alter the mRNA expression of markers associated with secretory lineage differentiation or goblet cell maturation (Figure 3H–J, *p* > 0.05). Thus, the increase in colonic goblet cell abundance following GOS supplementation was not accompanied by detectable activation of the canonical Wnt, BMP, or Notch pathway-related genes.

These findings indicated that the protective effect of GOS on goblet cell abundance was unlikely to be mediated by canonical intestinal stem cell proliferation or terminal goblet cell differentiation through the Wnt/BMP/Notch pathway.

### 3.4. Effect of GOS on CHOP and MAPK Signaling

Goblet cells synthesize and secrete large amounts of mucins, placing substantial demands on protein folding and processing within the endoplasmic reticulum (ER) [18,32]. We therefore first examined whether ER stress-associated apoptosis (CHOP pathway) contributed to AGEs-induced goblet cell depletion and whether GOS acted through the pathway.

**Figure 4:**
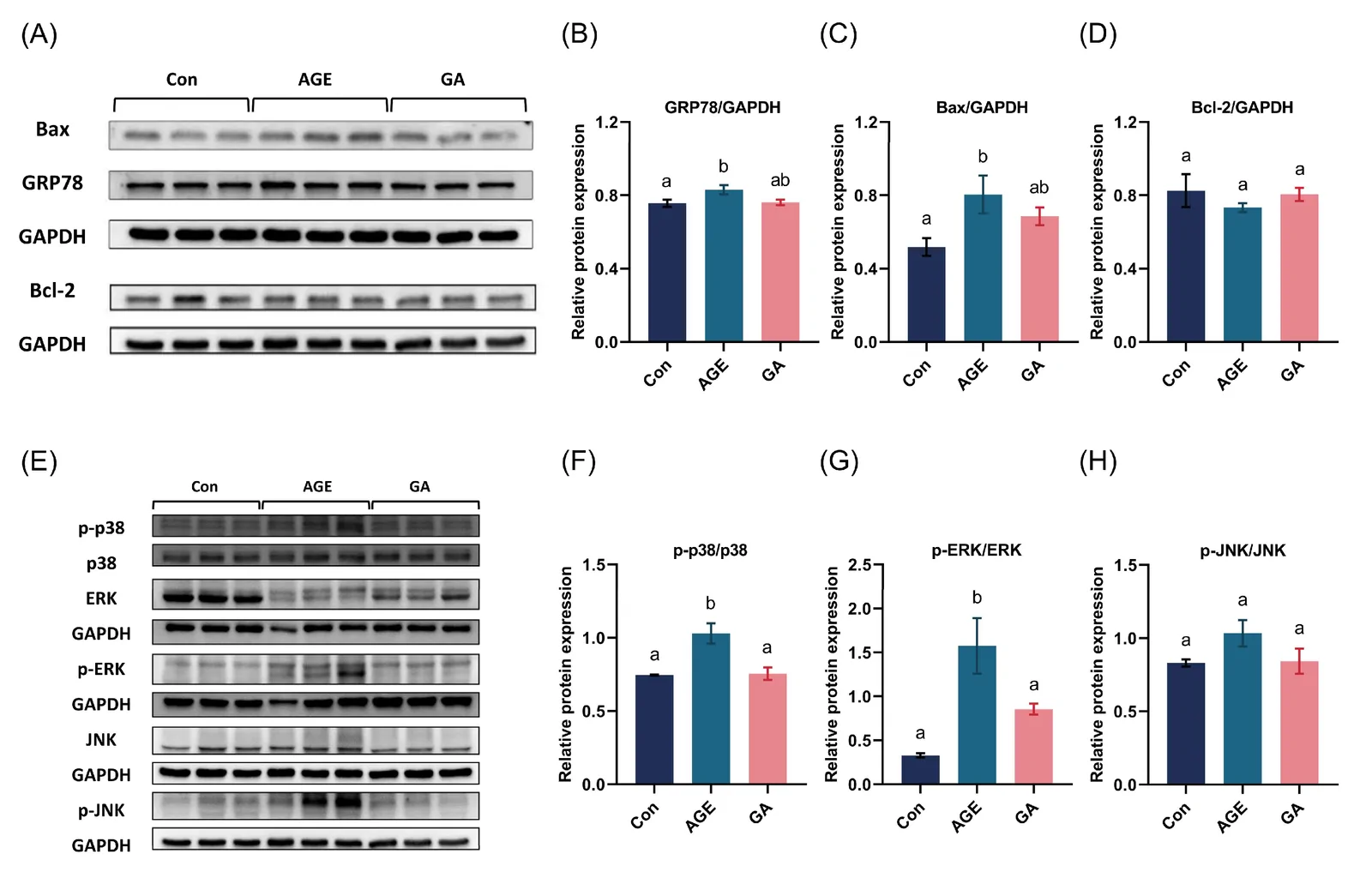
Effect of GOS on colonocyte apoptosis in mice fed with dietary AGEs. Western blot assays of Bax, GRP78, and Bcl-2 expression (**A**); relative GRP78 expression (**B**); relative Bax expression (**C**); relative Bcl-2 expression (**D**); Western blot assays of p-p38, p38, ERK, p-ERK, JNK, and p-JNK ratio (**E**); relative p-p38/p38 expression (**F**); relative p-ERK/ERK ratio (**G**); relative p-JNK/JNK ratio (**H**). Different letters represent significant differences (*p* < 0.05).

Compared with the Con group, the AGE group exhibited significantly increased levels of the ER stress marker GRP78 (9.21%, *p* < 0.05) and the pro-apoptotic protein Bax (53.85%, *p* < 0.05). However, GOS supplementation did not significantly reduce either GRP78 or Bax protein expression compared with the AGE group (*p* > 0.05). In addition, Bcl-2 expression did not differ significantly among the three groups (*p* > 0.05) (Figure 4A–D). These findings suggested that the protective effect of GOS on AGEs-induced goblet cell depletion was not mediated by detectable suppression of the CHOP-associated apoptotic response.

We next examined MAPK signaling, which is involved in regulating cellular stress responses and apoptosis [17]. The phosphorylation ratios of p38 and ERK were significantly elevated in the AGE group compared with the Con group, with increases of 37.33% and 378.79%, respectively (*p* < 0.05). GOS supplementation significantly reduced the ratios of p-p38/p38 and p-ERK/ERK by 26.21% and 46.20%, respectively, compared with the AGE group (*p* < 0.05). By contrast, the p-JNK/JNK ratio did not differ significantly among the three groups (*p* > 0.05) (Figure 4E–H).

Collectively, these results suggested that the preservation of goblet cell abundance by GOS coincided with suppression of AGEs-associated p38 and ERK activation, whereas the measured markers of the CHOP-associated ER stress pathway did not appear to account for this effect.

### 3.5. Effect of GOS on AGEs Receptor Expression

AGEs-RAGE signaling has been implicated in the activation of downstream MAPK pathways [33]; we next examined whether alterations in AGEs receptor expression were associated with the effects of dietary AGEs and GOS. We assessed the colonic mRNA expression of RAGE and the receptors associated with AGEs uptake and clearance (AGE-R1, AGE-R2, and AGE-R3).

**Figure 5:**
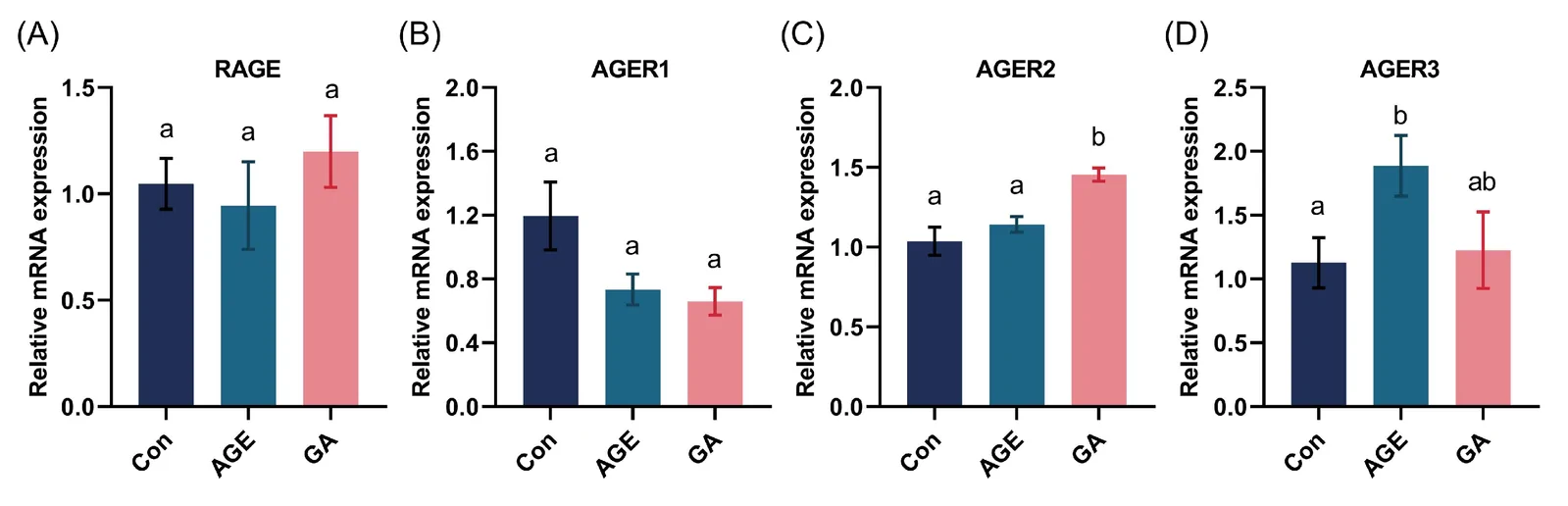
Effects of GOS supplementation on AGEs receptors in the colon of dietary AGEs-fed mice. The mRNA expression levels of RAGE (**A**), AGE-R1 (**B**), AGE-R2 (**C**), and AGE-R3 (**D**) in the colon tissues were investigated. Different letters represent significant differences (*p* < 0.05).

No significant differences in RAGE, AGE-R1, or AGE-R2 mRNA expression were observed between the Con and AGE groups (*p* > 0.05), whereas AGE-R3 mRNA expression was significantly increased in the AGE group compared with the Con group (*p* < 0.05). GOS supplementation significantly increased AGE-R2 mRNA expression compared with the AGE group (*p* < 0.05), but did not significantly alter RAGE, AGE-R1, or AGE-R3 mRNA expression (*p* > 0.05) (Figure 5A–D).

Thus, the protective effects of GOS on the colonic mucus barrier were not accompanied by changes in colonic RAGE mRNA expression. These findings do not support altered RAGE transcription as the primary explanation for the effects of GOS on MAPK signaling.

### 3.6. FMT Recapitulated the Effects of GOS on St3gal1 and ERK

**Figure 6:**
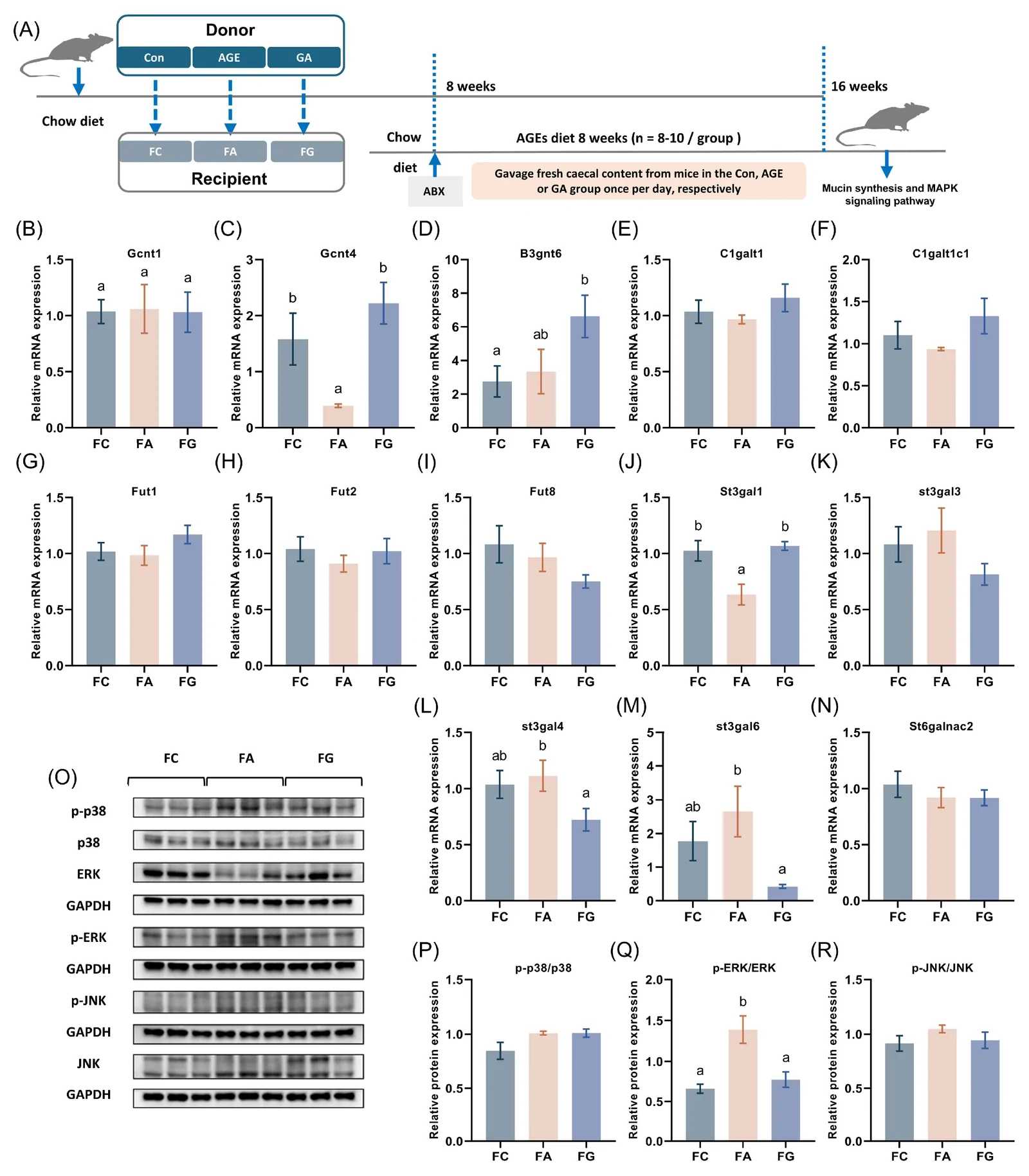
The expression of glycosyltransferases and the MAPK signaling pathway in the colon of FMT mice. Experimental design for FMT (**A**); the mRNA expression levels of Gcnt1 (**B**), Gcnt4 (**C**), B3gnt6 (**D**), C1galt (**E**), C1galt1c1 (**F**), Fut1 (**G**), Fut2 (**H**), Fut8 (**I**), St3gal1 (**J**), St3gal3 (**K**), St3gal4 (**L**), St3gal6 (**M**), and St6galnac2 (**N**) in the colon tissues were investigated; Western blot assays of p-p38, p38, ERK, p-ERK, JNK, and p-JNK expression (**O**); relative p-p38/p38 ratio (**P**); Relative p-ERK/ERK ratio (**Q**); relative p-JNK/JNK ratio (**R**). Different letters represent significant differences (*p* < 0.05).

Our previous study demonstrated that GOS supplementation alleviated AGEs-induced gut microbiota dysbiosis and that transplantation of fecal microbiota from GOS-treated mice partially restored the colonic mucus barrier in AGEs-fed recipient mice [4]. These findings raised the possibility that the microbiota may contribute to the regulation of mucin glycosylation and intracellular signaling. The experimental design of FMT is depicted in Figure 6A.

To investigate this possibility, we examined mucin glycosylation-related enzymes and MAPK signaling in recipient mice following FMT. No significant differences were observed between the FG and FA groups in the mRNA expression of Gcnt1, B3gnt6, C1galt1, C1galt1c1, Fut1, Fut2, Fut8, St3gal3, or St6galnac2 (*p* > 0.05). By contrast, St3gal1 expression was significantly reduced in the FA group compared with the FC group, whereas transplantation of microbiota from GOS-treated donors significantly increased St3gal1 expression compared with the FA group (*p* < 0.05) (Figure 6B–N). The direction of these changes was consistent with that observed in the corresponding donor groups.

We further assessed MAPK signaling in recipient mice. The FA group exhibited a significantly increased p-ERK/ERK ratio compared with the FC group, whereas this increase was significantly attenuated in the FG group (*p* < 0.05) (Figure 6O–R). This pattern was consistent with the findings obtained in the donor mice.

These findings support a partial, microbiota-dependent mechanism underlying GOS-mediated protection against dietary AGEs-induced intestinal mucus barrier dysfunction, whereby the gut microbiota may contribute to the modulation of St3gal1 mRNA expression and ERK signaling.

## 4. Discussion

The present study provided evidence for a potential mechanism that GOS protected the colonic mucus barrier against dietary AGEs-induced dysfunction through coordinated modulation of mucin glycosylation and intracellular signaling. Our findings indicated that the protective effect was not explained by restoration of the examined genes involved in mucin core O-glycan biosynthesis or activation of the canonical Wnt/BMP/Notch pathways involved in intestinal stem cell proliferation and goblet cell differentiation. Instead, GOS selectively increased the mRNA expression of the sialyltransferases St3gal1 and St3gal3 and attenuated AGEs-associated p38 and ERK activation. Importantly, FMT from GOS-treated donors reproduced the changes in St3gal1 mRNA expression and p-ERK/ERK signaling in recipient mice, suggesting that the gut microbiota contributed to the mucosal response to GOS. These findings collectively support the conclusion that GOS-associated microbiota changes were likely linked to mucin sialyltransferase St3gal1 and ERK signaling during protection against dietary AGEs-induced mucus barrier dysfunction.

In the colon, a gel-forming mucin secreted by goblet cells constitutes the mucous layer covering the colonic epithelium [5]. Mucin structurally resembles a bottle brush with a protein core at its center and glycans (at least 80% of the molecular mass) acting as bristles [27]. Mouse colonic mucin predominantly consists of Core2 structures [34], with GCNT1 and GCNT4 catalyzing the synthesis of these structures [35]. Our results showed that dietary AGEs significantly reduced the mRNA levels of GCNT1 and GCNT4 in the mouse colon, whereas no significant difference was observed in the mRNA levels of enzymes associated with Core1 (C1galt1 and C1galt1c1) and Core3 (B3gnt6) structures. These findings suggested that dietary AGEs may impair the transcriptional capacity for Core 2 O-glycan biosynthesis, potentially affecting the composition of mucin O-glycan structures in the colon. Our findings further suggested that the protective effects of GOS involve the selective regulation of mRNA expression associated with mucin terminal glycosylation. Although GOS did not restore the mRNA expression of core 2-associated glycosyltransferases, it significantly increased St3gal1 and St3gal3 mRNA expression in AGEs-fed mice. In addition to sialylation, mucin fucosylation represents another important form of terminal glycan modification [28]. Supplementation with GOS significantly down-regulated colonic Fut2 mRNA levels in dietary AGEs-fed mice. Although FUT2 loss-of-function variants have been associated with genetic susceptibility to IBD, increased FUT2 expression has also been reported in inflamed intestinal mucosa from patients with Crohn’s disease [27]. These findings suggested that the biological effects of FUT2 may depend on the disease context. Interestingly, mucin fucosylation in intestinal epithelial cells is regulated by interactions between the host and the gut microbiota. Schroeder [36] reported that the presence of intestinal microbiota induced expression of genes encoding Muc2 and Fut2, which affected mucus strength and mucin glycan structure. Furthermore, mucin quantity alone does not fully determine mucus barrier function; the composition and terminal modification of mucin glycans can influence mucus physicochemical properties and interactions with the intestinal microbiota [9,37]. The selective response of St3gal1, St3gal3, and Fut2 mRNA expression therefore suggested that GOS may modify the functional characteristics of the mucus layer through changes in terminal modification rather than by broadly restoring mucin O-glycan biosynthesis. However, our mRNA data only suggested potential transcriptional regulation of mucin glycosylation-related enzymes. Direct characterization of mucin glycan structures (e.g., mass spectrometry) would be required to confirm functional changes in mucin glycosylation, which represents a future research direction.

Previous studies have reported associations between oligosaccharide supplementation, St3gal1/St3gal3 expression, and gut microbiota composition [38]. Consistent with these observations, our FMT experiments revealed that transplantation of microbiota from GOS-treated mice significantly increased St3gal1 mRNA expression in AGEs-exposed recipient mice. However, St3gal3 mRNA expression was not similarly reproduced following FMT, indicating that regulation of mRNA expression associated with individual glycosyltransferases may involve distinct microbiota-dependent and host-dependent mechanisms. Further studies integrating recipient microbiome profiling, microbial metabolites, and functional manipulation of candidate taxa will be necessary to determine the precise molecular link between GOS-modulated microbiota and mucin glycosylation.

RAGE is a well-established receptor for AGEs and can activate downstream signaling pathways, including ERK/MAPK signaling [39,40]. However, our study did not detect significant changes in colonic RAGE mRNA expression following dietary AGEs exposure or GOS supplementation. Therefore, the present findings do not support altered RAGE transcription as the primary explanation for the changes in ERK phosphorylation observed in response to GOS. Importantly, the absence of changes at the mRNA level does not exclude a role for RAGE protein abundance, ligand binding, receptor activation, or downstream signaling. The discrepancy between our in vivo findings and previous in vitro studies reporting AGEs-induced RAGE upregulation in Caco-2 cells may partly reflect differences in experimental models [35]. In vivo, dietary AGEs encounter the mucus layer before reaching the epithelial surface, whereas conventional epithelial cell models lack this physiological mucus interface [5]. Thus, future studies using mucus-producing epithelial co-culture systems and direct assessment of RAGE protein activation will be necessary to clarify whether RAGE contributes to AGEs-induced ERK activation.

An important finding of the present study was that the molecular effects associated with GOS supplementation were partially reproduced by FMT. Our previous study showed that transplantation of microbiota from GOS-treated mice improved the colonic mucus barrier in AGEs-exposed recipients [4]. Here, we extend these observations by showing that FMT also restored St3gal1 expression and reduced the p-ERK/ERK ratio in recipient mice. The concordance between donor and recipient phenotypes suggested the concept that the gut microbiota contributes to the regulation of mucin glycosylation and mucosal signaling during GOS-mediated protection. However, we did not perform 16S rRNA sequencing or other microbiome profiling in the recipient mice used in the present FMT experiments. Therefore, we cannot determine which microbial taxa or microbial functions were responsible for the observed changes, nor can we establish a direct causal relationship between specific microbiota alterations, St3gal1 expression, and ERK signaling. Additionally, FMT inocula are prepared as suspensions of donor feces in sterile saline, and the supernatant is collected for administration. The possible co-transfer of residual GOS or microbial metabolites cannot be excluded; however, this study did not quantify the concentration of residual GOS or fermentation metabolites in the FMT preparations. Consequently, the biological effects observed following fecal microbiota transplantation cannot be attributed solely to the transfer of the microbiota itself; the potential co-transfer of soluble metabolites, such as residual GOS from the supernatant of donor feces, constitutes a confounding factor. Future studies combining longitudinal microbiome profiling with microbial metabolomics and targeted microbial or pharmacological interventions will be important for resolving this microbiota-mucin glycosylation/ERK axis.

## 5. Conclusions

In conclusion, GOS alleviated dietary AGEs-induced colonic mucus barrier dysfunction potentially involving selective regulation of mucin glycosylation-related gene expression and suppression of MAPK signaling. Specifically, GOS increased the mRNA expression of St3gal1 and St3gal3 and attenuated AGEs-associated p38 and ERK activation, whereas the canonical Wnt/BMP/Notch pathways and the CHOP-associated apoptotic pathway did not appear to account for the protective effect. FMT partially recapitulated the effects of GOS on St3gal1 expression and ERK activation, supporting a contributory role of the gut microbiota. Collectively, these findings identify mucin glycosylation and ERK signaling as potential molecular components of the protective response to GOS during dietary AGEs exposure and provide a mechanistic framework for understanding how prebiotic intervention may preserve colonic mucus barrier function.

## Figures and Tables

**Table 1:** Primers used for qPCR.

Primers	Forward Sequence (5′-3′)	Reverse Sequence (5′-3′)
Gcnt1	ACAGATTCAGGCTTCCTGTGATT	GCCAGGTGAGATGCCAGTTTA
Gcnt4	TACGAGCACGAGCCTTTGG	AGGGTCTGATAAACGTCACAGT
B3gnt6	GGCCAGATTCTCCTCTCTCAAAC	CAGTGTCGTGGGACTCTTGAAC
C1galt1	ATGGACACAGTCACCTCAAAGG	GAGGTTCTCAGCAACGTCTATGT
C1galt1c1	TCTCACGTCCAAGCCTCGT	TGTGGCCTAGCATAGTGATCAAG
Fut1	AGAATTCGCTTGCACCACCA	AAGAAGGAGCCGGCAGAGA
Fut2	TGAACTTTCGGCTAAGGTACATCT	GGAAGTGGGCCAGAGGAAAG
Fut8	AGGCGAATGGCTGAGTCTCT	TGGCCTTAACAAGCTGTTCTTCT
St3gal1	GGTTCCCCAAGCAGATGCTC	CCAATATGATACCTTGTCCTGGC
St3gal3	TGCTGCGGTCATGTAGGAAA	CAGCGGAGTCAAGGGAAAGA
St3gal4	GGCTCTGGTCCTTGTTGTTG	TCCCTAGAACGGTTGCCAAAA
St3gal6	CACCCCAAAAGCGCAGATTTATT	CCTGCCTGAAACAGAGTCCAA
St6galnac2	CGGATGTTGTTGCTCGTTGC	AGTCGGCTCTTTCTGTTTTCC
Gpr81	AGCCCTAGTGTTCGGATAGGCA	AAGCAGAAGCCGCACAGGG
Wnt3	CTTCTAATGGAGCCCCACCT	GAGGCCAGAGATGTGTACTGC
Lgr5	CGGCAACAGTGTGGACGACCT	GCGAGCACTGCACCGAGTGA
Ki67	CCTGCCCGACCCTACAAAAT	TTGCTCACACTCGATGCAGT
BMP2	AGCTGCAAGAGACACCCTTT	CATGCCTTAGGGATTTTGGA
BMP4	ATTGGCTCCCAAGAATCATGG	CGTGATGGAAACTCCTCACAGT
Smad1	CTCACATGCCACTGAACGCC	TCCGCATACACCTCTCCTCC
Smad5	TTTGCAGAGCCATCACGAGC	TTTCCAATATGCCGCCTAGT
Smad8	CACCGACCCTTCCAATAAC	CTGGACAAAGATGCTGCTG
Notch1	ACAGTAACCCCTGCATCCAC	GGTTGGACTCACACTCGTTG
Notch2	GACTGCACAGAAGACGTGGA	GCGTAGCCCTTCAGACACTC
Notch3	GTGTCAATGGTGGTGTCTGC	GCACACTCATCCACATCCAG
Notch4	GAGGGACACTCCACCTTTCA	CTGGTGCCTGACACAGTCAT
Jagged1	CCAGCCAGTGAAGACCAAGT	CAATTCGCTGCAAATGTGTT
Hes1	GCGAAGGGCAAGAATAAATG	TGTCTGCCTTCTCTAGCTTGG
Math1	CAAGTGTGTCCAGCAGTGTG	TTGAGTTTCTTCAAGGCGGC
Klf4	GTGCCCCGACTAACCGTTG	GTCGTTGAACTCCTCGGTCT
Elf3	CCTATGAGAAGCTGAGCCGA	ACCTCTTCTTCCTTCCAGCC
Spdef	GGAGAAGGCAGCATCAGGA	CCAGGGTCTGCTGTGATGT
RAGE	ACTACCGAGTCCGAGTCTACC	CCCACCTTATTAGGGACACTGG
AGE-R1	GCTGGACAACCTGAACGTG	TCCACCGACGGGGAAAAGA
AGE-R2	ACCTTTCACCTGTTTGGACGG	GTCGCAGTAGTCGTCGTTCA
AGE-R3	AGACAGCTTTTCGCTTAACGA	GGGTAGGCACTAGGAGGAGC
β-actin	CACTGTCGAGTCGCGTCC	TCATCCATGGCGAACTGGTG

**Table 2:** Primary antibodies used in Western blot.

Primary Antibody	Brand and Catalog Number	Dilution
Bax	Wanlei (WL03419)	1:1000
GRP78	Wanlei (WL03157)	1:2000
Bcl-2	Wanlei (WL01556)	1:500
JNK	Wanlei (WL01295)	1:1000
p-JNK	Wanlei (WL01813)	1:1000
p38	Wanlei (WL00764)	1:1000
p-p38	Wanlei (WL03482)	1:500
ERK1/2	Wanlei (WL01864)	1:500
p-ERK1/2	Wanlei (WL01512)	1:300
GAPDH	Huaan (ET1601-4)	1:5000

## Data Availability

The original contributions presented in this study are included in the article/Supplementary Materials. Further inquiries can be directed to the corresponding author.

## Supplementary Materials

The following supporting information can be downloaded at: https://www.mdpi.com/article/10.3390/foods15213806/s1. Figure S1: Effect of GOS on the expression of the Wnt pathway in the colon of dietary AGEs-fed mice. The mRNA expression levels of Gpr81 (A), Wnt3 (B), Lgr5 (C), and Ki67 (D) in the colon tissues were investigated. Figure S2: Effect of GOS on the expression of the BMP pathway in the colon of dietary AGEs-fed mice. The mRNA expression levels of BMP2 (A), BMP4 (B), Smd1 (C), Smd5 (D), and Smd8 (E) in the colon tissues were investigated.

## Abbreviations

The following abbreviations are used in this manuscript:

AGEs	advanced glycation end products
GOS	galacto-oligosaccharides
GCs	goblet cells
FMT	fecal microbiota transplantation
ZO-1	zonula occludens-1
RAGE	receptor for advanced glycation end products
